# In Memoriam

**Published:** 1887-11

**Authors:** 


### In Memoriam.

Died June 22nd, 1887, in Bay St. Louis, Miss., Dr. J. R. Walker, D.D.S., of nervous prostration, in connection with some chronic affections of long standing, which were due in a good degree to confinement and arduous professional labor, in years gone-by.

Dr. Walker was born in Thompkins county, N. Y., August

7th, 1830, of progenitors distinguished for intelligence, learning, and integrity. His father's family was of Maryland stock, and his mother of New England lineage.

In 1834 the family moved to the State of Michigan where this son acquired the rudiments of a good education. His seventeenth year was spent in teaching and studying in Illinois. Here he began the study of dentistry, which he continued the following year at Albion, Michigan.

The next year he completed his course of professional study with Dr. Foster, of Jackson, Michigan. After this, instead of entering at once into the practice of his profession, he visited eastern cities, in order that he might become familiar with the best modes of practice, and with the best instruments and appliances that were available.

In 1854 he went to the State of Texas where he conducted a very successful practice for four years. In 1858 he removed to New Orleans, where he soon established a very successful business.

In 1865 he married Miss Jeaie Mort, a lady of English parentage, of culture, and of education. Mrs. Walker is well-known in the dental profession as a ready and fluent writer, her "Letters to Mothers," written a few years ago, is the best thing extant on that subject.

Dr. Walker was always very active in what he conceived to be of interest to his profession ; in it he occupied many positions of trust and honor, and was always faithful to every responsibility imposed upon him. The writer, in an acquaintance of over thirty years with him, always found him the same genial, affable, pleasant gentleman.

Outside of his profession he took a great interest in various matters of science ; indeed almost every branch of science had an attraction for him ; and as a result he was a man of broad culture and attainments, both in literature and science. He will ever be remembered by those who knew him best, as a true friend, and as a strong advocate of that which he conceived to be right ; and impatient with, and antagonistic to that which he thought to be wrong.

The bereaved ones have the warm sympathy of a host of friends and acquaintances.

